# Transcriptome analysis reveals the effect of cold storage time on the expression of genes related to oxidative metabolism in Chinese black truffle

**DOI:** 10.3389/fnut.2024.1375386

**Published:** 2024-06-04

**Authors:** Runji Zhang, Qiuyue Yang, Xin Yao, Zhirong Fang, Xia Wu, Qiao Lin, Yuan Qing

**Affiliations:** ^1^Key Laboratory of Panxi Featured Crops Research and Utilization, Xichang University, Xichang, China; ^2^College of Agricultural Sciences, Xichang University, Xichang, China; ^3^College of Resources and Environment, Xichang University, Xichang, China

**Keywords:** *Tuber indicum*, Chinese black truffle, post-harvest storage, transcriptome, oxidative metabolism, simple sequence repeat

## Abstract

Chinese black truffle (*Tuber indicum*) is a hypogenous fungus of great value due to its distinctive aroma. In this study, both transcriptome and physicochemical analyses were performed to investigate the changes of nutrients and gene expression in truffle fruiting bodies during cold storage. The results of physicochemical analysis revealed the active metabolism of fruiting bodies in cold storage, showing the decreased contents of protein and soluble sugar, the variations in both polyphenol oxidase activity and total phenol content, and the detrimental effect of reactive oxygen species production caused by heavy metals (cadmium and lead) in truffles. Transcriptome analysis identified a total of 139,489 unigenes. Down-regulated expression of genes encoding the catalase-like domain-containing protein (katE), glutaredoxin protein (GRX), a copper/zinc superoxide dismutase (Sod_Cu), and aspartate aminotransferase (AAT) affected the degradation metabolism of intracellular oxides. Ribulose-5-phosphate-3-epimerase (RPE) was a key enzyme in response to oxidative stress in truffle cells through the pentose phosphate pathway (PPP). A total of 51,612 simple sequence repeats were identified, providing valuable resources for further genetic diversity analysis, molecular breeding, and genetic map-ping in *T. indicum*. Transcription factors GAL4 and SUF4-like protein were involved in glucose metabolism and histone methylation processes, respectively. Our study provided a fundamental characterization of the physicochemical and molecular variations in *T. indicum* during the cold storage at 4°C, providing strong experimental evidence to support the improvement of storage quality of *T. indicum*.

## 1 Introduction

Truffles are the fungal species in the genus *Tuber* (Ascomycota, Pezizales) producing hypogenous fruiting bodies (1, 2), which are commonly known as treasured and delectable food due to their distinctive scent (3). The commercial truffles in China mainly belong to *T. indicum* complex, which is occasionally referred to as the Chinese black truffle or Asian black truffle, with obvious morphological features such as presence of nodules and black surface of the fruiting body; these truffles are closely related to *T. melanosporum*, sharing many physicochemical characteristics (1, 4). Truffles are well known as food abundant in proteins, fatty acids, carbohydrates, amino acids, and minerals (5, 6). As the seasonal products, truffles could only maintain high quality and sensory characteristics for a relatively short time after harvest, while the market demand for fresh truffles could be prolonged and met only by proper preservation techniques, which have attracted increasing attention (7).

Generally, fresh food deteriorates during cold storage as observed in the loss of flavor and color and development of off-putting odors, caused by structural changes and growth of microbes (8). Previous studies investigated the optimal preservation methods of four popular truffles, i.e., *T. magnatum*, *T. borchii*, *T. melanosporum*, and *T. aestivum*, revealing that the most beneficial effects on the biochemical and microbial parameters of truffles were observed under 4°C cold storage (9). The truffle fruiting bodies generally maintain a low metabolic activity, and cold storage could help maintain the low metabolic activity and delay spoilage in truffles, although the metabolic activity could not be completely terminated. As a result, the proteins and carbohydrates as well as the texture of the fruiting bodies continue to change. For example, studies have shown that some edible fungi, such as *Pleurotus tuoliensis* and *Agaricus bisporus*, are degraded after cold storage, i.e., loss of weight, turning brown, softening fruiting bodies, wilted, lidding, and invasion by microbes (10, 11). Similarly, studies on the browning level of *Coprinus comatus* and *Lentinus edodes* in storage showed that cold storage could not completely maintain the texture quality of edible fungi, ultimately affecting the commercial value of the products (12, 13). Furthermore, the amounts of soluble proteins and carbohydrates in *Pleurotus eryngii* are considerably decreased after 12 days of storage, whereas the amount of total free amino acids is increased, indicating that the carbohydrates and soluble proteins are the essential resources to support the metabolic functions during the postharvest storage of edible fungi, and the levels of these essential resources are rapidly decreased with the deterioration of fruiting bodies (10). These results are in agreement with those previously reported, showing that *Flammulina velutipes* suffered severe browning and lost its market value after 15 days of storage at 4°C (14).

Studies have shown that the preservation period of both *T. melanosporum* and *T. aestivum* could be extended by a combined method of modified atmosphere packaging (MAP) and cold storage, with the main purpose of inhibiting respiration and transpiration of fruiting bodies (15). For example, it was reported that the combination of both MAP and cold storage extended the freshness period of *T. matsutake* from 10 to 18 days (16). Although these results indicate that the physicochemical properties of edible fungi are changed during postharvest storage and the occurrence of these changes could be delayed by various methods, the molecular mechanisms underlying these variations are still unclear. Considering the high economic value of *Tuber indicum*, their fruiting-bodies are extremely prone to spoilage during postharvest transportation and storage. In order to evaluate the effect of storage time on the quality of the fruiting-bodies of *T. indicum*, three storage times (0, 15, and 30 days) were selected under the conventional storage temperature (4°C). On the one hand, we would like to identify the optimal time for cold chain transportation. On the other hand, it is important to explore the variations in both physiochemical properties and molecular characteristics of truffles at different times, in order to identify and develop appropriate strategies to decrease the deterioration of truffles during storage. In particular, the identification of genes involved in oxidative metabolism of truffle during cold storage could provide novel molecular evidence to support the improvement of storage technology, e.g., molecular engineering of corresponding enzymatic agents to prevent the aging and deterioration processes based on the genetic components involved in oxidative metabolism or improving the molecular breeding of truffle varieties with enhanced storage efficiency after harvest.

To date, studies on the molecular explorations of *T. indicum* cold storage are sparse due to the lack of a completely sequenced genome or transcriptome (17). It is necessary to perform the high-throughput investigations of variations in both transcription and ex-pression of genes involved in the changes of physicochemical properties of truffes during postharvest storage (18, 19). Therefore, the *de novo* transcriptome analysis was performed in this study to explore the molecular mechanisms regulating the biological processes involved in the alterations of physicochemical properties of *T. indicum* during different cold storage stages. The goals of our study were: (1) to reveal the effect of cold storage time on the expression of genes related to oxidative metabolism in *T. indicum*, (2) to characterize the physicochemical and molecular variations in *T. indicum* during the cold storage at 4°C, and (3) to provide experimental evidence to support the development of truffle preservation technologies and further investigation of the molecular and physiological mechanisms underlying the physicochemical variations of *T. indicum* after harvest.

## 2 Materials and methods

### 2.1 Collection of *Tuber indicum* samples

Samples of truffles used in this study were collected from the field of Huidong County, Sichuan, China (E 102°20′-103°3′, N 26°12′-26°55′), with the sporophores hand-picked from the ground. The taxonomic identification of the specimens used in our study was determined as the Chinese black truffle, which belonged to the *Tuber indicum* complex, mainly based on the available references (1, 4), using evident characters, e.g., visible black surface and the obvious tuberosity. The entire sporophores of similar sizes were then selected, wrapped in aluminum foil, placed in a low-temperature storage box with ice packs, and then promptly transferred to the laboratory. The truffles were washed three times with sterile water to remove the surface soil, dried on an ultra-static table, and then transferred into a sterile bag for storage in the 4°C refrigerator. Three groups of samples were prepared in 0 d (the control group ZD), 15 d (the experimental group FD), and 30 d (the experimental group TD) of cold storage, respectively, and were surface sterilized with 75% alcohol. A small piece of tissue (cube of about 1 cm^3^) was collected from the interior of the fruiting body using a sterile razor blade performed on an alcohol-sterilized work surface, transferred to 1.5 mL sterile Eppendorf tubes, and kept at –80°C for transcriptome analysis and at 4°C for physicochemical property examination, respectively. Each experiment was repeated with three or more biological replicates.

### 2.2 Physicochemical property determination of *Tuber indicum* samples

A total of 10 physiological, biochemical and physicochemical properties, including the respiration rate, polyphenol oxidase (PPO) activity, polysaccharide content, protein content, total organic content (TOC), total phenol content (TPC), and four texture properties (hardness, resilience, elasticity, and chewability), were determined in three groups of fruiting body samples. TOC was determined by potassium dichromate volumetric method and the protein content was determined by Kjeldahl method (20, 21). TPC was determined by the Folin-Ciocalteu assay with minor modifications and the content of polysaccharide was determined by sulfuric acid-phenol method (22, 23). PPO activity of the *T. indicum* buffer solution was evaluated with UV spectrophotometer (TU-1810, Puxi, Beijing, China) using the method previously reported (24). The respiration rate of the truffle was determined by measuring the amount of oxygen absorbed or carbon dioxide released (25, 26). Both corruption rate and weight loss rate were determined as previously described (27). The contents of cadmium (Cd) and lead (Pb) were measured according to the method previously reported (28). The quantitative determination of elements was performed using the Inductively Coupled Plasma Mass Spectrometry (ICP-MS) Series (iCAP RQ; Thermo Scientific, USA). Each of the above experiments was repeated with three biological replicates.

### 2.3 RNA extraction and transcriptome sequencing

Trizol Reagent (Invitrogen Life Technologies, USA) was used to extract total RNA, with the integrity and purity of the RNA determined using the Agilent Bioanalyzer 2100 (Agilent Technologies Inc., Santa Clara, CA, USA) and the RNA quantified by Nano-Photometer^®^ spectrophotometer (IMPLEN, CA, USA). The library was sequenced on the Novaseq 6000 platform (Illumina) at Wekemo Tech Group Co. Ltd., Shenzhen, China. FastQC (v0.11.9; default parameters) software was used for data quality control, with requirements of Q30 ≥ 80% to ensure the high performance of subsequent analyses. Trinity (v2.1.1.; default parameters) (accessed on 02 March 2022)^1^ was used to splice the clean data of transcriptome based on *T. indicum* samples for the *de novo* assembly due to the lack of reference genome of this taxon. iTAK (v1.6; default parameters;^2^ accessed on 02 March 2022) was used for prediction of transcription factors (TFs). The simple sequence repeat (SSR) loci from transcriptome sequence were identified based on transcriptome analysis using the MIcroSAtellite (MISA) software (Microsatellite, v1.0; default parameters;^3^ accessed on 02 March 2022). The raw data were submitted to National Center for Biotechnology Information (NCBI) database (accession number PRJNA952816).^4^ The functions of genes of *T. indicum* transcriptome were annotated using the non-redundant (NR) protein sequence and the Nucleotide Sequence (NT) database of NCBI, the SwissProt database,^5^ the Uniprot database,^6^ the Kyoto Encyclopedia of Genes and Genomes (KEGG) database,^7^ and the Gene Ontology (GO) database,^8^ respectively (29).

### 2.4 Identification of differentially expressed genes

Clean reads of *T. indicum* transcriptome were mapped to unigenes assembled by StringTie (v1.3.3b) using a reference-based approach (30). TPM, the expected number of Transcripts Per Kilobase of Exon Model per Million mapped Reads Sequenced, is currently the most popular method for measuring the gene expression levels, simultaneously taking into account the influence of both sequencing depth and gene length for the read count (31). Differential expression analysis of three groups (each group with four biological replicates) of fruiting body samples was performed using the DESeq2 R package (version 1.16.1), with statistical methods for identifying differential expression based on gene expression data using a negative binomial distribution-based model (32). Differentially expressed genes (DEGs) were identified using DESeq2 based on | log_2_(Fold Change)| ≥ 1 and adjusted *P* value of 0.05 (33).

### 2.5 Functional annotation and metabolic pathway enrichment analysis of DEGs

The DEGs identified in the *T. indicum* transcriptome were further annotated using GO database (see text footnote 8; accessed on 07 March 2022) to identify GO terms in three categories, i.e., cellular component, molecular function, and biological process, respectively. Genome annotation was based on Ensembl and KEGG (Kyoto Encyclopedia of Genes and Genomes). For pathway enrichment analysis, DEGs were mapped into KEGG pathways using the KOBAS 2.0 (accessed on 07 March 2022).^9^ The GO and KEGG analysis based on *P* value less than 0.05 were considered significantly enriched by DEGs.

### 2.6 Quantitative real-time PCR validation

A total of 20 DEGs were randomly selected for quantitative real-time PCR (qRT-PCR) validation, which was performed on the CFX96 Real-Time System (BIO-RAD) with SYBR green used as the fluorescent dye. Each experiment was repeated with three biological replicates, using 18S rRNA as an internal reference gene (34). The relative expression of genes was calculated using the 2^–ΔΔCt^ method (35). Differential expression of genes was considered statistically significant using Duncan test based on P < 0.05.

### 2.7 Statistical analysis

Statistical analysis was performed using IBM SPSS Statistics version 26 (IBM Corporation, New York, USA). Data were presented as mean ± standard deviation (SD). The significant differences among groups were analyzed using one-way ANOVA, followed by Duncan test to determine the significant differences based on *P* < 0.05.

## 3 Results

### 3.1 Physicochemical characteristics of *Tuber indicum*

**FIGURE 1:**
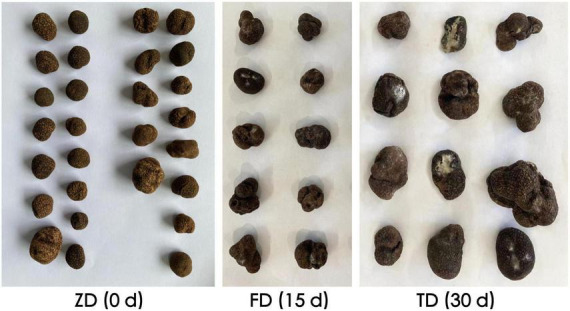
Morphological variations observed in three groups of *Tuber indicum* samples in 0 d (ZD), 15 d (FD), and 30 d (TD) of cold storage at 4°C, respectively.

Evident variations in morphological features such as mildew and rot were observed in truffle fruiting bodies due the 30-d cold storage (Figure 1). The physicochemical properties of three groups of *T. indicum* samples were evaluated (Table 1). In 30 d of cold storage at 4°C, the respiration rate of *T. indicum* was decreased from 271.12% to 101.77%, whereas the corruption rate and weight loss rate were gradually increased as the storage time was increased. The contents of both protein content and soluble sugar were increased as the storage time was increased from 0 d (group ZD) to 30 d (group TD), reaching the maximum levels of 13.98% and 8.67%, respectively, in the TD group. Similarly, both TOC and TPC of TD group were higher than those of ZD group, while the PPO activity was decreased as the storage time was increased, suggesting that the longer storage time caused the decrease in PPO activity of *T. indicum*. As the storage time was increased, the content of Cd was decreased in three groups of samples in the order of ZD > TD > FD, while the content of Pb was significantly increased in 30 d of cold storage compared with the control group.

**TABLE 1:** Physicochemical characteristics of *Tuber indicum* in 0 d (ZD), 15 d (FD), and 30 d (TD) of cold storage at 4°C.

Property	ZD (0 d)	FD (15 d)	TD (30 d)
Respiration rate (%)	271.12 ± 1.03^a^	198.71 ± 2.28^b^	101.77 ± 1.75^c^
Protein content (%)	21.35 ± 2.78^a^	17.37 ± 1.05^b^	12.61 ± 0.62^c^
Soluble sugar (mg/g)	14.47 ± 0.27^a^	11.34 ± 0.45^b^	9.08 ± 0.09^c^
TOC (mg/g)	408.40 ± 1.01^b^	433.82 ± 0.53^a^	434.39 ± 1.03^a^
TPC (mg/g)	4.01 ± 0.01^b^	4.54 ± 0.03^a^	4.59 ± 0.04^a^
PPO (U)	19.78 ± 0.53^a^	13.72 ± 0.87^b^	5.72 ± 0.39^c^
Corruption rate (%)	0	6.98 ± 1.98^b^	13.98 ± 1.35^a^
Weight loss rate (%)	0	3.40 ± 0.23^b^	8.67 ± 0.77^a^
Content of cadmium (mg/kg)	1.05 ± 0.05^a^	0.70 ± 0.02^c^	0.88 ± 0.03^b^
Content of lead (mg/kg)	1.23 ± 0.11^b^	1.10 ± 0.02^b^	1.84 ± 0.05^a^

Data are presented as average ± SD (*n* = 3). Different superscript letters a, b, and c in the same row denote statistically significant differences (*P* < 0.05). TOC, total organic content; TPC, total phenol content; PPO, polyphenol oxidase.

### 3.2 RNA-seq analysis and de novo assembly of transcriptome of Tuber indicum

A total of 40,001,150 to 45,136,560 raw reads were generated based on a total of 12 libraries of three groups of *T. indicum* samples in 0, 15, and 30 d of cold storage at 4°C, respectively (Supplementary Table 1). After removing the reads of lower quality, a total of 9,834,325 to 40,807,628 clean reads were obtained. The Q20, Q30, and GC contents indicated that these sequencing libraries were reliable. A total of 268, 599 transcripts were acquired using Trinity and grouped into a total of 169,932 unigenes, which were produced in the meta-assembly, with the lengths ranging between 201 and 23,955 bp with an average of 3,428 bp (Supplementary Table 1).

### 3.3 Functional annotation of genes of *Tuber indicum*

**FIGURE 2:**
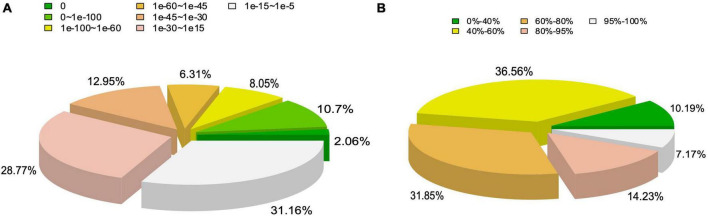
Statistics of functional annotation of genes derived from *Tuber indicum* transcriptome based on non-redundant protein database of NCBI. **(A)** E-value distribution. **(B)** Sequence similarity distribution.

The functional annotation of the unigenes identified by the transcriptome analysis was further performed using BLASTx with a cut-off E-value < 10^–5^. The results showed that 58.16% of the 169,932 unigenes were annotated by at least one database (Supplementary Table 2). Among the annotated genes, 31.16% of the matches were detected with *E*-values ranging from e^–15^ to e^–5^ (Figure 2A), while 67.41% of the unigenes showed a similarity ranging between 40% and 80%, and 21.4% of the unigenes showed a similarity higher than 80% with the accessible fungal gene sequences (Figure 2B).

**FIGURE 3:**
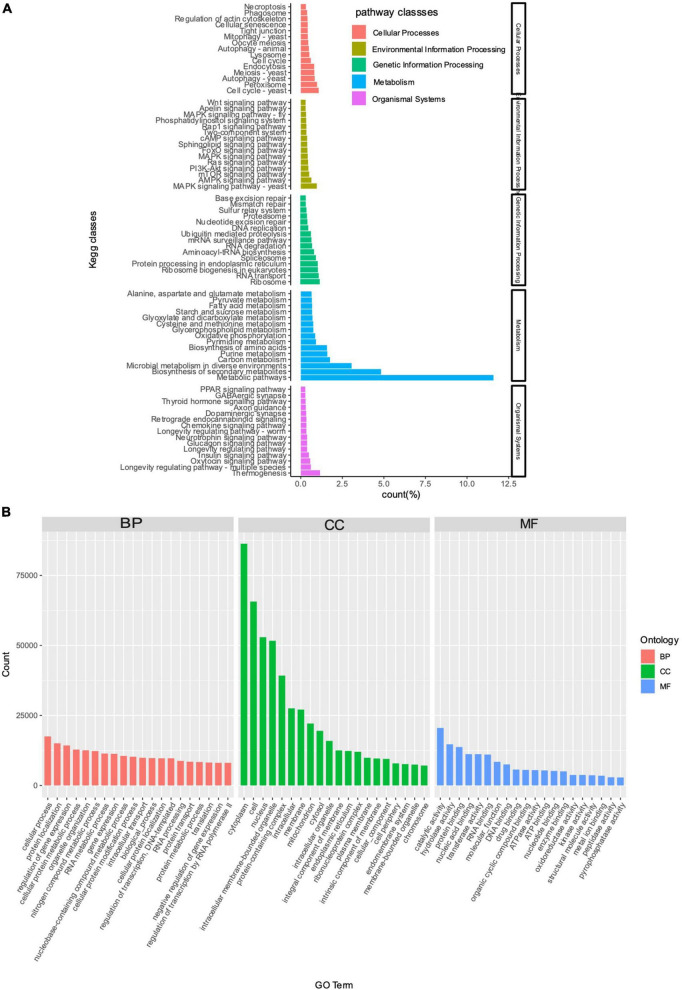
KEGG enrichment **(A)** and GO annotation **(B)** analyses based on unigenes derived from the transcriptome of *Tuber indicum* under cold storage at 4°C. DEGs are enriched in five classes of KEGG metabolic pathways (i.e., cellular processes, environmental information processing, genetic information processing, metabolism, and organismal systems) and three categories of GO terms, i.e., BP, biological processes; CC, cellular component; MF, molecular function, respectively.

The results of KEGG enrichment analysis revealed that a total of 25,358 (14.92%) unigenes of *T. indicum* were annotated in the KEGG database (Supplementary Table 2). The annotated unigenes were assigned to a total of five classes of metabolic pathways, including me-tabolism (31.11%), followed by genetic information processing (10.52%), environmental information processing (6.52%), cellular processes (8.97%), and organismal systems (6.46%) (Figure 3A). The top three significantly enriched pathways included metabolic pathways, biosynthesis of secondary metabolites, and microbial metabolism in diverse environ-ments, all belonging to the metabolism class of KEGG metabolic pathways.

The results of GO annotation analysis showed that a total of 22,100 (13.01%) unigenes of *T. indicum* were annotated in the GO database (Supplementary Table 2), including 217,244 sequences annotated in the GO term category of biological processes, 504,115 sequences annotated in the category of cellular components, and 151,257 sequences annotated in the category of molecular functions (Figure 3B). In the category of biological processes, the top three GO terms enriched with the highest number of sequences included “cellular process” (17,512, 7.74%), “protein localization” (15,084, 6.94%), and “regulation of gene expression” (14,288, 6.58%); in the category of cellular components, the top four GO terms were cytoplasm” (86,340, 17.13%), “cell” (65,702, 13.03%), “nucleus” (52,944, 10.50%), and “intracellular membrane-bounded organelle” (51,081, 10.25%); the GO term “catalytic activity” was enriched with the most sequences in the category of molecular functions (20,556, 13.59%) (Figure 3B; Supplementary Table 3).

### 3.4 Transcriptomics response of *Tuber indicum* to different storage times

The TPM analysis was performed to assess the transcriptome responses of *T. indicum* to different cold storage times (Supplementary Figure 1A), revealing that the gene expression levels were similar among the three different groups of truffle samples, which could be used for the subsequent differential expression analysis of genes. The results of principal com-ponent analysis (PCA) based on all unigenes identified in *T. indicum* at different storage times showed that the three groups of samples were clearly distinctive from each other (Supplementary Figure 1B). These findings revealed the alterations in *T. indicum* transcriptome and the differential gene expression patterns in *T. indicum* at different storage times.

A total of 1,342 TFs were identified in a total of 43 families based on the Plant Transcription Factor Database (Table 2),^10^ with the C2H2 family having the largest number of transcripts (348, 25.93% of all TFs), followed by the zn-clus family with a total of 271 transcripts (20.19%). In the TD group (30-d storage), a total of 9 DEGs (3 up-regulated and 6 down-regulated) and 15 DEGs (8 up-regulated and 7 down-regulated) were annotated into the C2H2 and zn-clus families, respectively, compared with control group (0 d) (Supplementary Figure 2). Moreover, two down-regulated genes of the zn-clus family, *Cluster-3341.52929* and *Cluster-3341.54461*, encoded a putative nitrogen assimilation transcription factor nit-4 protein (GAL4). In the C2H2 family, four down-regulated genes (i.e., *Cluster-3341.80689*, *Cluster-3341.30934*, *Cluster-3341.35233*, and *Cluster-3341.48569*) encoded a C2H2-type zinc finger transcription factor (SUF4-like).

**TABLE 2:** Family and number of copies of transcription factors (TFs) predicted in *Tuber indicum* under cold storage at 4°C.

TF family	Count	Percent (%)
C2H2	348	25.93
zn-clus	271	20.19
C3H	94	7.00
bZIP	93	6.93
SNF2	42	3.13
SET	41	3.06
GNAT	35	2.61
HMG	33	2.46
bHLH	29	2.16
C2C2-GATA	28	2.09
TRAF	27	2.01
PHD	26	1.94
MYB-related	25	1.86
MED7	21	1.56
Others	229	17.06
Total	1342	100

### 3.5 Expression analysis of DEGs

**FIGURE 4:**
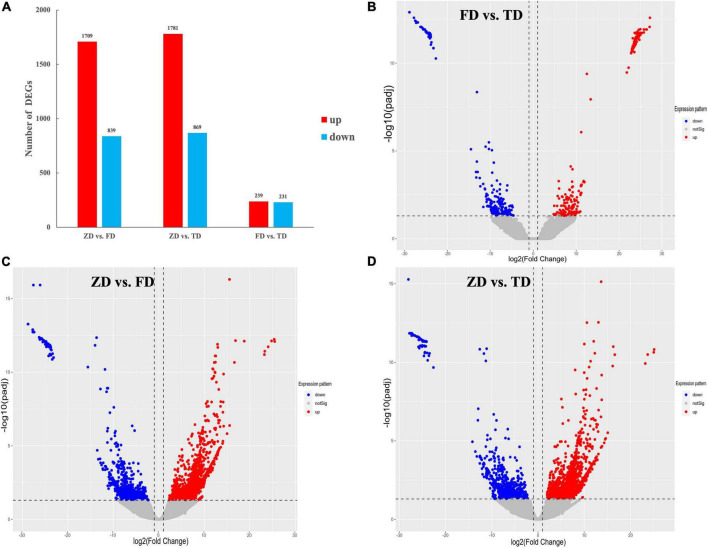
Differentially expressed genes (DEGs) identified among three groups of *Tuber indicum* samples, i.e., 0 d (ZD), 15 d (FD), and 30 d (TD) of cold storage at 4°C, respectively **(A)**, showing the volcano plots of DEGs between FD vs. TD **(B)**, ZD vs. FD **(C)**, and ZD vs. TD **(D)**, respectively. DEGs are determined based on | log2(Fold Change)| ≥ 1 and *P* < 0.05.

The expression of unigenes identified in all three groups of *T. indicum* was normalized based on the group of ZD, with the DEGs identified based on | log2(Fold Change) | ≥ 1 and P < 0.05. A total of 2,548 DEGs (1709 up-regulated and 839 down-regulated) were detected between the ZD and FD groups, and a total of 470 DEGs (239 up-regulated and 231 down-regulated) were identified between the FD and TD groups (Figure 4). Compared to the control group (ZD), a total of 2,650 DEGs were identified in the TD group (1,781 up-regulated and 869 down-regulated). The substantially large numbers of DEGs identified indicated that gene expression was significantly altered in the fruiting bodies of truffles during cold storage. The numbers of up-regulated DEGs were greater than those of down-regulated DEGs in FD vs. ZD and TD vs. ZD groups, while similar numbers of both up-regulated and down-regulated DEGs were identified in FD vs. TD groups. These results suggested that a variety of physiological processes were tapered off from 15 to 30 d of cold storage in the fruiting bodies of *T. indicum*.

**FIGURE 5:**
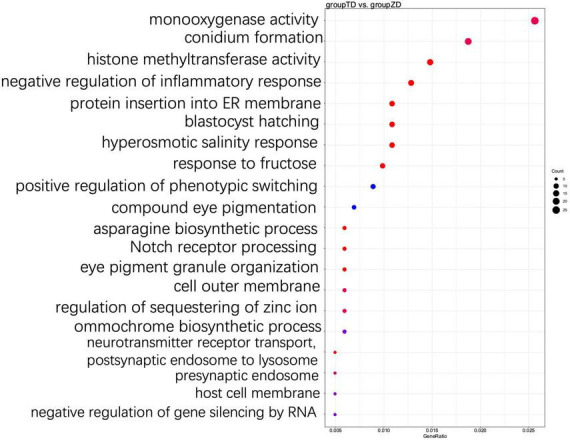
Top 20 most significantly enriched GO terms based on GO annotation of differentially expressed genes (DEGs) between TD (30-d cold storage) and ZD (control, 0-d cold storage) groups of *Tuber indicum* at 4°C.

The functions of DEGs were further explored using GO annotation analysis (Figure 5). The results showed that in the GO term category of biological processes, a group of five GO terms, i.e., asparagine biosynthetic process, protein insertion into ER membrane, response to fructose, hyperosmotic salinity response, and blastocyst hatching, were significantly enriched in the TD group, while the top three GO terms with the highest number of DEGs enriched included terpenoid biosynthetic process (GO:0016114; 14 up-regulated and 10 down-regulated), cellular oxidant detoxification (GO:0098869; 5 up-regulated and 13 down-regulated), and cellular response to reactive oxygen species (ROS) (GO:0034614, 3 up-regulated and 19 down-regulated) (Supplementary Table 4). The results showed that a total of 10 genes (*Cluster-3341.103909*, *Cluster-3341.48292*, *Cluster-3341.62642*, *Cluster-3341.81520*, *Cluster-3341.81521*, *Cluster-3341.84546*, *Cluster-3341.85532*, *Cluster-3341.120447*, *Cluster-3341.49143*, and *Cluster-3341.76098*) were down-regulated in the two GO terms associated with ROS, and encoded the catalase-like domain-containing protein (katE, KAG0138173.1) in cells, containing the similar domain as that of hydroperoxidase. In particular, both *Cluster-3341.120447* and *Cluster-3341.49143* encoded the glutaredoxin protein (GRX, KAG0127770.1), which was involved in cellular defense against oxidative stress, and *Cluster-3341.76098* encoded a copper/zinc superoxide dismutase (Sod_Cu, KAG0125302.1). In the GO term category of cellular component, a total of 60 DEGs were annotated into 12 GO terms related to cell membrane and cell wall components. In the GO term category of molecular functions, DEGs were largely enriched in histone methyltransferase activity, monooxygenase activity, peroxidase activity, and oxidoreductase activity in the TD group (Supplementary Table 4); in particular, a total of 15 DEGs (6 up-regulated and 9 down-regulated) were enriched in histone methyltransferase activity and histone methylation, and a total of 6 DEGs associated with asparagine biosynthetic process were all up-regulated. Among these six genes, both *Cluster-3341.99658* and *Cluster-3341.99659* encoded the pyridoxal phosphate-dependent transferase (KAG0135316.1) (Supplementary Table 4).

**FIGURE 6:**
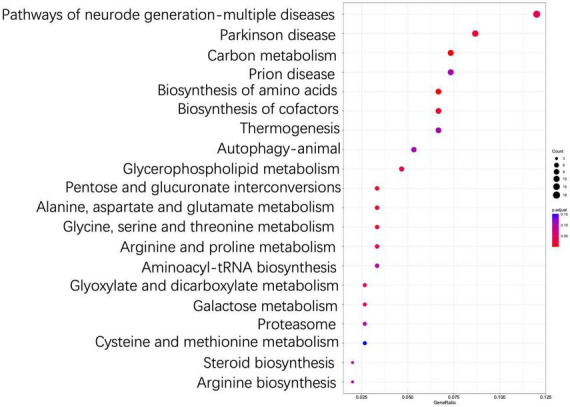
Top 20 most significantly enriched KEGG pathways based on differentially expressed genes (DEGs) between TD (30-d cold storage) and ZD (control, 0-d cold storage) groups of *Tuber indicum* at 4°C.

**FIGURE 7:**
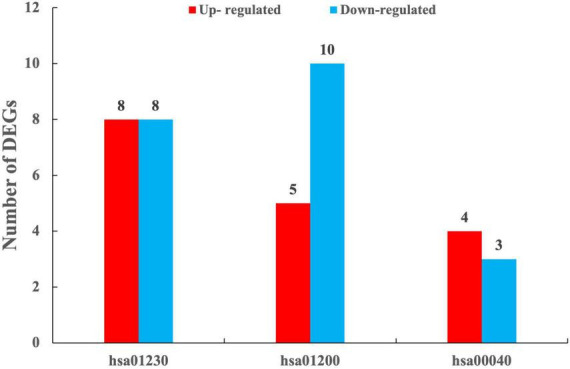
Numbers of differentially expressed genes (DEGs) enriched in the metabolic pathways of biosynthesis of amino acids (hsa01230), carbon metabolism (hsa01200), and pentose and glucuronate interconversions (hsa00040) between TD (30-d cold storage) and ZD (control, 0-d cold storage) groups of *Tuber indicum* at 4°C.

The results of KEGG enrichment analysis showed that DEGs were significantly enriched into a total of five categories of the metabolic pathways, i.e., metabolism, cellular processes, organismal systems, genetic information processing, and environmental information processing (Supplementary Table 5). These results indicated that *T. indicum* refrigerated at 4°C for 30 d was revealed to show the greatest impact on the metabolic activities, especially on biosynthesis of amino acids, carbon metabolism, biosynthesis of cofactors, pentose and glucuronate interconversions, the alanine, aspartate and glutamate metabolism, and the glycine, serine and threonine metabolism pathways (*P* < 0.05) (Figure 6, Supplementary Table 6). In addition, the metabolic pathways of chemical carcinogenesis-reactive oxygen species, peroxisome, fatty acid metabolism, and proteasome in *T. indicum* were also significantly affected by cold storage for 30 d (*P* < 0.05) (Supplementary Table 6). DEGs enriched in biosynthesis of amino acids, carbon metabolism, and biosynthesis of cofactors pathways showed the highest degree of enrichment and significance. A total of 8 up-regulated and 8 down-regulated DEGs were enriched in the biosynthesis of amino acids pathway (hsa01230), 4 and 3 genes were up-regulated and down-regulated in the pentose and glucuronate interconversions pathway (hsa00040), respectively, and 5 and 10 genes were up-regulated and down-regulated in the carbon metabolism pathway (hsa01200), respectively (Figure 7). It was noted that the up-regulated gene *Cluster-3341.50639* (KAG0635590.1) appeared in all three of these metabolic pathways. In the carbohydrate metabolic pathway, this gene encoded the ribulose-5-phosphate 3-epimerase (RPEase: EC 5.1.3.1), which played both racemase and epimerase activities to act on carbohydrates and their derivatives.

### 3.6 Validation of DEGs by qRT-PCR

The relative expression levels of a total of 20 DEGs, e.g., four genes annotated to histone methyltransferase activity, nine enriched in cellular oxidative metabolism, and two annotated to asparagine biosynthetic process, were verified by qRT-PCR analysis (Table 3). The results showed that the expression patterns of 17 out of 20 genes were consistent with those of RNA-seq analysis (Table 3).

**TABLE 3:** Validation by qRT-PCR of a total of 20 differentially expressed genes (DEGs) identified by transcriptome analysis of fresh *Tuber indicum* in 30 d of cold storage at 4°C.

GO term	Gene ID	RNA-seq		qRT-PCR	
		Log_2_(FC)	*P* value	Log_2_(FC)	*P* value
Histone methyltransferase activity	Cluster-3341.68849	25.13	0.00	21.47	0.01
	Cluster-3341.88222	−8.29	0.01	−9.63	0.01
	Cluster-3341.68156	−7.70	0.02	−7.15	0.03
	Cluster-3341.76233	11.79	0.00	10.85	0.02
	Cluster-3341.71662	8.95	0.01	8.42	0.01
Asparagine biosynthetic process	**Cluster-3341.66828**	**–12.86**	**0.00**	**–10.59**	**0.10**
	Cluster-3341.71275	−13.77	0.00	−15.13	0.03
	Cluster-3341.99659	-4.13	0.00	−4.01	0.01
Monooxygenase activity	Cluster-3341.66023	5.94	0.01	6.19	0.02
	Cluster-3341.29554	−2.62	0.04	−1.94	0.01
	Cluster-3341.63320	6.75	0.00	6.26	0.01
	Cluster-3341.68147	−7.09	0.03	−8.39	0.02
Terpenoid biosynthetic process	Cluster-3341.126477	7.84	0.01	8.03	0.03
	Cluster-3341.53123	3.51	0.01	3.97	0.01
	Cluster-3341.64515	−8.13	0.01	−9.24	0.02
	**Cluster-3341.71614**	**12.00**	**0.00**	**14.33**	**0.13**
Cellular response to reactive oxygen species	Cluster-3341.63673	5.57	0.02	5.81	0.01
	Cluster-3341.72904	6.08	0.02	5.62	0.04
	**Cluster-3341.103909**	**–2.60**	**0.00**	**–1.91**	**0.12**
	Cluster-3341.96576	−11.16	0.00	−10.80	0.01

Three genes not verified by qRT-PCR are highlighted in bold. Data represent the variations in the value of Log_2_(FC) based on truffles in group TD (30 d under cold storage) relative to those of group ZD (0 d under cold storage). FC, fold change.

### 3.7 Simple sequence repeat analysis

All the 169,932 unigenes based on transcriptome were mined for microsatellites or putative genic simple sequence repeats (genic-SSRs). The results revealed varied number and density distribution of different types of SSRs detected in a total of 51,612 SSR motifs identified in *T. indicum* (Table 4), with the highest number of SSRs detected as mono-nucleotide repeats (p1, 73.40%), followed by tri-nucleotide (p3, 11.50%), di-nucleotide (p2, 8.61%), tetra-nucleotide (p4, 5.14%), penta-nucleotide (p5, 0.98%), and hexa-nucleotide motifs (p6, 0.36 %). The di-nucleotide repeat AG/CT (of a total of 2,621 SSRs) was the most prevalent type of SSRs, followed by other di-nucleotide (1,825 SSRs) and tri-nucleotide repeats (5,936 SSRs) (Supplementary Table 7).

**TABLE 4:** Distribution of the total number of SSRs in six different classes based on the length of basic unit of SSRs (i.e., p1 to p6) identified in *Tuber indicum* under cold storage at 4°C.

Class of SSR	Number (%)
p1: mono-nucleotide	37,884 (73.40)
p2: di-nucleotide	4,446 (8.61)
p3: tri-nucleotide	5,936 (11.50)
p4: tetra-nucleotide	2,654 (5.14)
p5: penta-nucleotide	508 (0.98)
p6: hexa-nucleotide	184 (0.36)

## 4 Discussion

### 4.1 Effect of cold storage on physicochemical properties of *Tuber indicum*

Although the fruiting bodies of *T. indicum* are rich in nutrients, e.g., carbohydrates and proteins, their qualitative and sensory properties are only retained for a short time after harvest (7, 8). Previous studies mainly focused on the volatile substances and antioxidant activities of *T. indicum* in storage, as well as the relationship between truffles and microorganisms (8, 22, 36). In our study, the variations of physicochemical properties and the contents of two heavy metals (Cd and Pb) of truffles during cold storage were investigated. The results showed that the respiration rate, protein content, soluble sugar content, and PPO activity in truffle fruiting bodies were gradually decreased as the storage time was increased, reaching the lowest levels in 30 d of cold storage, whereas the corruption rate, weight loss rate, TOC, and TPC were increased as the storage time was increased. These results indicated that the physicochemical properties were altered during the cold storage, largely consistent with the results previously reported (8, 22, 36).

### 4.2 Quality change of *Tuber indicum* in cold storage caused by Cd and Pb

It has been reported that many truffles have become contaminated in their natural environments with heavy metals, such as Cd, chromium, and Pb, as a result of the rapid growth of industrialization, ultimately causing serious risk for human health (36, 37). Our results showed that in 30 d of cold storage, the content of Cd was decreased, whereas the content of Pb was significantly accumulated. Furthermore, previous studies revealed detrimental effects of exposure to heavy metals on the levels of proteins (38). Studies have shown that the soluble protein content is a crucial indicator of both reversible and irreversible changes in metabolism, which is well known to react with a variety of endogenous and exogenous stressors (39). It has been reported that the oxidative metabolism of cells is affected by the content of heavy metals, and the presence of heavy metals will lead to the production of reactive oxygen species in cells to further damage cells (40). We note that the truffles used in our study are commonly consumed locally and nationally, and no incidence of heavy metal contamination or poisoning has been reported on these fungal taxa. Moreover, studies have indicated that heavy metals precipitate in the formation of cell walls or chemical compounds to chelate with proteins and ultimately involve in the activities of fungal cell walls and the enzymatic activities in vivo (40–42). Therefore, based on the findings revealed in our study, it was speculated that the accumulation of Pb in the storage process of truffles could reduce the activities of cell walls and inhibit the activities of antioxidant enzymes. In addition, the toxicity of heavy metals could be caused by the production of ROS, damaging proteins, nucleic acids, and lipids, and ultimately leading to cell death, suggesting that heavy metals could enhance the process of truffle decay (43). Previous studies reported that higher PPO activity would lead to phenolic oxidation and browning reaction of mushrooms during storage (44). Our results showed that the PPO activity was decreased with the extension of storage time, whereas no significant difference was detected in TPC between 15 d and 30 d of cold storage, probably due to the cell aging or even cell death caused by the oxidative damage of ROS to proteins and other physiological processes, leading to the abnormal process of phenol oxidation and the accumulation of TPC during storage, as previously reported (45). Interestingly, as the storage time was increased, the contents of protein and soluble sugar of truffles were decreased, while the TOC was increased, suggesting that fresh fruiting bodies still underwent metabolic activities, attracting a large number of airborne spoilage microorganisms to accelerate the decomposition of these substances, as previous studies reported (46).

### 4.3 Effect of cold storage time on expression of genes associated with oxidative metabolism of *Tuber indicum*

Studies have shown that plants respond to adverse stress by regulating the expression of related genes in the process (47). For example, the histone methylation modification is related to the expression of genes involved in various biological processes and stress responses, i.e., the SET Domain Group (SDG) gene family encoding histone methyltransferase are involved in the regulation of the biological processes (48, 49). Furthermore, it has been reported that histone methylation plays a key role in plant response to temperature changes through dynamic variations in different histone methyltransferases in *Arabidopsis thaliana*, including the regulation of flowering time (50). To date, the post-translational modification of histones has gradually become one of the research hotspots in the field of microbiology. Studies have shown that the combination of post-translational modification of histones and environmental stress could affect the changes of fungal phenotype and material metabolism (51, 52). In our study, after 30 d of cold storage, the transcriptome analysis of truffle fruiting bodies revealed significantly enhanced activity of histone methylation process and expression of genes encoding histone methyltransferases, suggesting that the histone methyltransferase played an important role in response to low temperature stress in truffle fruiting bodies.

Due to the detrimental effects of free radicals on cellular constituents, such as lipids, proteins, and DNA, oxidative stress is presumably closely associated with the ripening and aging processes of fruits (53). Studies have shown that the association between ROS and cellular antioxidant mechanisms governs the crucial process of REDOX balance in cells (54). Our results showed that the TPC of fruiting bodies of *T. indicum* under cold storage gradually declined as the storage time was increased in groups ZD (21.35%), FD (17.37%), and TD (12.61%), respectively. Furthermore, the large number of DEGs associated with the oxidative metabolism of substances and enzymes were identified in truffles under cold storage for 30 d, suggesting the active breakdown of proteins, amino acids, and other nutrients in the fruiting bodies during this process. Moreover, in the TD group, the dominant enzymatic activities of monooxygenase, peroxidase, and oxidoreductase were regulated by DEGs, which mainly encoded the following enzymes or proteins: putative phenol 2-monooxygenase, endo-1,3(4)-beta-glucanase, glycosyltransferase family 20-domain-containing protein, catalase-like domain-containing protein, fatty acid synthase beta subunit dehydratase, and thioredoxin-like protein.

The results of this study showed that the expression of genes encoding katE protein, GRX protein, and Sod_Cu were down-regulated during the generation of ROS, and both katE protein and Sod_Cu were involved in the degradation and metabolism of intracellular oxides (55), while GRX protein was involved in cellular oxidative stress defense (56, 57), indicating that the expression of these genes of *T. indicum* stored at 4°C for 30 d was probably inhibited, resulting in the accumulation of oxides in truffle cells, causing oxidative damage to truffle fruiting body, and accelerating aging and deterioration processes. Furthermore, studies have shown that membrane dysfunction and stress disorders of agricultural products during cold storage are mainly caused by the peroxidation of structural lipids due to oxidative free radicals (58). These results were consistent with the findings revealed in our study, showing that peroxidation in *T. indicum* cells led to an increase in the level of peroxides, while the expression of peroxidase genes was inhibited, ultimately causing the accumulation of peroxides in truffle fruiting bodies. Moreover, our result showed that the expression of gene encoding pyridoxal phosphate-dependent transferase was up-regulated, which was a member of the aspartate aminotransferase (AAT) family. Previous studies showed that the gene encoding glutamic oxaloacetic aminotransferase (GOT) in *Ganoderma lucidum* was involved in the alterations of amino acid content, metabolism of carbohydrate and protein, and the production of ROS (59), while the degradation activity of *Serrella marcescens* was closely related to the up-regulation of succinyl-diaminopimelate transaminase (60). These studies indicated that the oxidative degradation of truffle fruiting bodies was closely related to the up-regulation of genes encoding the enzymes in the AAT family.

The results of KEGG enrichment analysis based on DEGs showed that the expression of genes encoding ribulose-phosphate 3-epimerase (RPEase: EC 5.1.3.1) was up-regulated in three metabolic pathways, i.e., biosynthesis of amino acids, pentose and glucuronate interconversions, and carbon metabolism. This enzyme was similar in structure to triose phosphate isomerase (TIM) and was a key enzyme of the pentose phosphate pathway (PPP). Studies have shown that the PPP plays a key role in maintaining NADPH/NADP homeostasis and provides protection against oxidative stress through ROS detoxification, and RPE enzymes were involved in the reversible conversion of D-ribulose 5-phosphate into D-xylulose 5-phosphate (61). Our results showed that after 30 d of cold storage, the cells of *T. indicum* were detected with oxidative stress response under the influence of low temperature environment, and the expression of genes encoding RPE in PPP was up-regulated, suggesting that RPE enzyme was probably the main molecular component involved in response to oxidative metabolism, ultimately exhibiting the protective effect of truffle cells themselves. These results were consistent with those previously reported, showing that the deficiency of RPE was related to susceptibility to oxidative stress in *Saccharomyces cerevisiae* (62) and that RPE was considered the main target of oxidative stress caused by hydrogen peroxide in *Escherichia coli* (63).

### 4.4 Prediction of SSR markers and transcription factors

SSR markers are frequently employed in functional genomics, association mapping, biodiversity analysis, genome mapping, transferability and comparative mapping, and marker-assisted selective breeding (64, 65). Although there are variations detected among individuals, the sequences of SSRs are generally conservative. The molecular polymorphisms based on SSRs have shown significant values for the genetic studies of biological stress resistance and marker-assisted molecular breeding (66, 67). For example, SSR markers were used to identify loci associated with soybean Phytophthora resistance, and SSR markers associated with blast resistance and sheath blight resistance were detected in a total of 91 rice cultivars (68, 69). In recent years, SSR markers have also been used in the genetic analysis of various edible fungal taxa, such as *Lentinus edodes*, *Flammulina velutipes*, and *Auricularia auricula* (70–72). In our study, a total of 96,871 SSRs were detected in truffle fruiting bodies, providing abundant molecular markers for further research on stress resistance and molecular breeding of truffles.

TFs bind DNA in a sequence-specific way to control gene transcription (73). Under stress conditions, organisms activate RNA polymerase II transcription complexes by stimulating TFs through a sequential signal transmission with the appropriate cis-acting element combination, activating the expression of particular genes and response to environmental signals (74, 75). Previous studies showed that TFs in association regulated the expression of functionally associated genes under stress conditions, and the overexpression of these TFs either stimulated or suppressed the expression of resistance-related genes to ultimately alter an organism’s capacity to survive in stressed environments (76). For example, studies have confirmed that a group of TFs, including bHLH, bZIP, MYB, C2H2, ERF, NAC, and WRKY, play an important role in plant responses to low temperature stress (75). Our results showed that a total of 1,342 TFs of 43 families were differentially expressed in truffles under cold storage for 0, 15, or 30 d, respectively, with the highest level of abundance of TFs revealed in the C2H2 family, followed by zn-clus, C3H, and bZIP families of TFs. In particular, our results showed that the gene encoding the GAL4 protein in the zn-clus TF family was down-regulated, in accordance with the results previously reported, showing that GAL4 protein was a positive regulator of the expression of genes involved in fungal glucose metabolism (77). These results suggested that GAL4 protein played an important role in the glucose metabolism of truffles, ultimately causing the phenotypic variations in the truffles under cold storage. Furthermore, our results showed that a total of 4 genes encoded the zinc finger domain protein (i.e., SUF4-like protein) of the C2H2 TF family. Due to the shared functional domain with *Oryza sativa* SUF4 (OsSUF4) and *Arabidopsis thaliana* SUF4 (AtSUF4), which regulated methyltransferase in the methylation process of proteins (78–80), it was speculated that SUF4-like protein was involved in regulating the expression of histone methylation-related genes in truffle fruiting bodies. In summary, the dynamic changes in the expression of genes encoding these TFs indicate that TFs play an important role in the storage process of truffles and are also associated with biological responses induced by temperature changes during the storage. Future investigations are necessary to further explore the molecular significance of these TFs and their regulatory effects on expression of genes involved in truffle response to cold temperatures.

## 5 Conclusion

The studies on the molecular mechanisms underlying the storage of *T. indicum* are significantly limited by the lack of completely sequenced genome or transcriptome. We applied de novo transcriptome sequencing to investigate the quality changes of *T. indicum* under cold storage at 4°C for 0 d (group ZD), 15 d (group FD), and 30 d (group TD), respectively, to reveal the effect of cold storage time on the expression of genes related to oxidative metabolism in *T. indicum*. We further characterized the physicochemical and molecular variations in *T. indicum* during the cold storage at 4°C. The results showed that the metabolic processes of the fruiting bodies of *T. indicum* in the cold storage were closely related to the quality changes of the truffles. The catabolism of carbohydrates and proteins in the truffle fruiting bodies was enhanced as the time of cold storage was increased, as observed in the variations of respiration rate, weight loss rate, and corruption rate. In particular, heavy metals such as Pb and Cd accumulated in *T. indicum* fruiting bodies were involved in the spoilage of truffles, and the changes of PPO and TPC in cells caused by heavy metals indicated that ROS production during storage could greatly damage the fruiting bodies of *T. indicum*. Transcriptome analysis showed that genes involved in oxidative metabolism (i.e., biosynthesis of amino acids, carbon metabolism, and pentose and glucuronate interconversions) were significantly enriched in truffles under cold storage at 4°C for 30 d. Down-regulated expression of genes encoding katE, GRX, Sod_Cu, and AAT affected the degradation metabolism of intracellular oxides. RPE was a key enzyme involved in response to oxidative stress in truffle cells through PPP. Transcription factors GAL4 and SUF4-like protein were involved in glucose metabolism and histone methylation processes, respectively, during the cold storage of truffles. In summary, the physiochemical variations in truffles under cold storage significantly impacted the nutritional value and mouthfeel of truffles, suggesting that in addition to the influence of environmental factors, storage techniques that caused the variations in related enzymes inhibiting the accumulation of oxides or enhancing the enzymatic activity of related antioxidant kinases should also be considered in the cold storage at 4°C of truffles. Our study provided strong experimental evidence to support the development of truffle preservation technologies in order to improve the quality of truffles under storage and further investigation of the molecular and physiological mechanisms underlying the physicochemical variations in *T. indicum* after harvest.

## Data availability statement

The datasets presented in this study can be found in online repositories. The names of the repository/repositories and accession number(s) can be found in the article/Supplementary material.

## Author contributions

RZ: Methodology, Software, Visualization, Writing−original draft, Writing−review and editing. QY: Formal analysis, Validation, Methodology, Project administration, Writing−review and editing. XY: Methodology, Validation, Writing−review and editing. ZF: Investigation, Writing−review and editing. XW: Resources, Investigation, Writing−review and editing. QL: Formal analysis, Methodology, Investigation, Writing−review and editing. YQ: Conceptualization, Funding acquisition, Project administration, Resources, Writing−review and editing.

## Footnotes

1
http://trinityrnaseq.sourceforge.net/

2
http://itak.feilab.net/cgi-bin/itak/index.cgi

3
http://pgrc.ipk-gatersleben.de/misa/

4
https://www.ncbi.nlm.nih.gov/sra/

5
https://www.ebi.ac.uk/uniprot/

6
https://www.uniprot.org/

7
https://www.genome.jp/kegg/

8
https://geneontology.org/

9
https://kobas.cbi.pku.edu.cn/

10
http://planttfdb.cbi.pku.edu.cn/
