# Supplementary material for: Transcriptome analysis reveals the effect of cold storage time on the expression of genes related to oxidative metabolism in Chinese black truffle
Source: Front Nutr. 2024 Jun 4;11:1375386. doi: 10.3389/fnut.2024.1375386 (PMC11183293; doi:10.3389/fnut.2024.1375386)
Supplement: Supplementary file 1 [file Image_1.pdf]

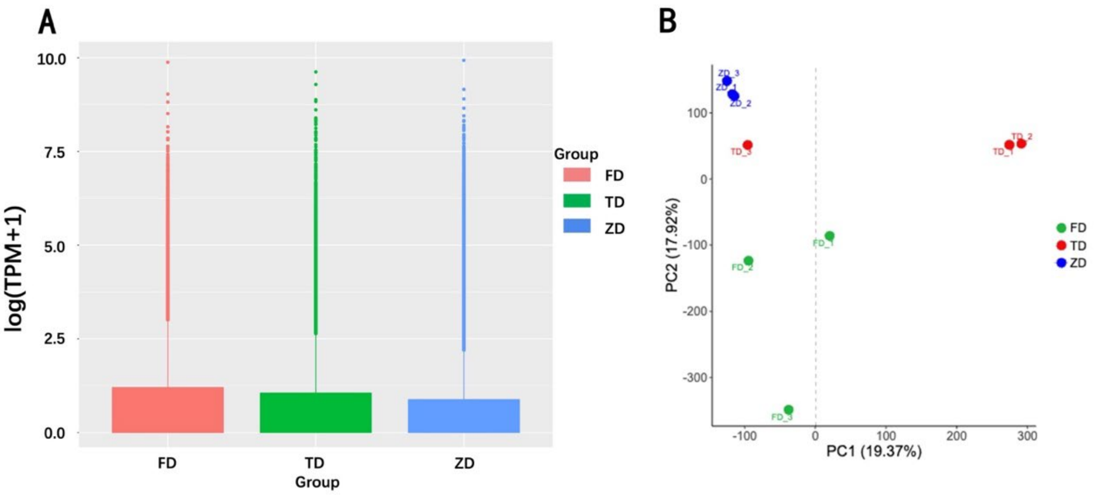


**Figure S1.** TPM analysis (**A**) and principal component analysis (**B**) based on transcriptome investigation of *Tuber indicum* under different cold storage times, i.e., 0 d (ZD), 15 d (FD), and 30 d (TD), respectively.

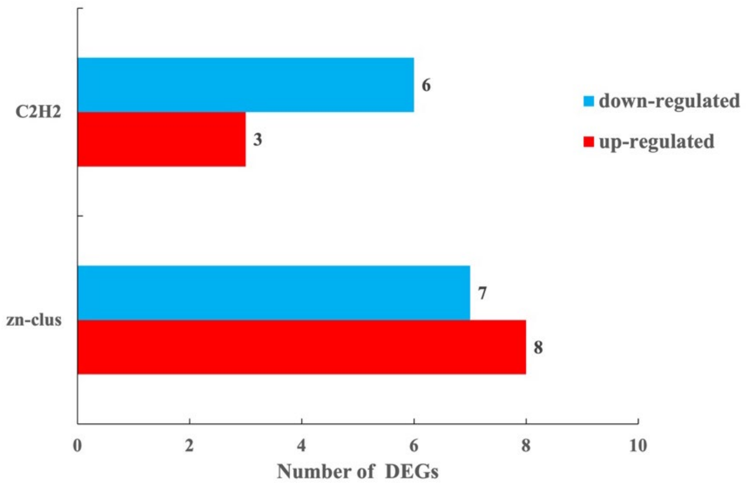


**Figure S2.** Numbers of differentially expressed genes (DEGs) involved in two transcription factor families (C2H2 and zn-clus) between TD (30-d storage) and ZD (0-d storage; control) groups of *Tuber indicum* at 4 °C.
