# Supplementary material for: Transcriptome analysis reveals the effect of cold storage time on the expression of genes related to oxidative metabolism in Chinese black truffle
Source: Front Nutr. 2024 Jun 4;11:1375386. doi: 10.3389/fnut.2024.1375386 (PMC11183293; doi:10.3389/fnut.2024.1375386)
Supplement: Supplementary file 2 [file Table_1.docx]

**Table S1.** Statistics of transcriptome sequencing of *Tuber indicum* under different cold storage time, i.e., 0 d (ZD), 15 d (FD), and 30 d (TD), respectively.

| **Sample** | **Raw read** | **Clean read** | **Clean base (G)** | **Q20 (%)** | **Q30 (%)** | **GC content (%)** |
| --- | --- | --- | --- | --- | --- | --- |
| ZD1 | 40,903,524 | 37,678,950 | 5.52 | 100 | 99.88 | 50 |
| ZD2 | 41,592,172 | 38,499,458 | 5.64 | 100 | 99.89 | 50 |
| ZD3 | 45,136,560 | 9,834,325 | 1.43 | 100 | 99.89 | 50 |
| ZD4 | 41,860,796 | 38,547,904 | 5.65 | 100 | 99.89 | 50 |
| FD1 | 40,001,150 | 36,875,118 | 5.4 | 100 | 99.89 | 50 |
| FD2 | 44,249,000 | 40,782,898 | 5.98 | 100 | 99.89 | 50 |
| FD3 | 44,162,374 | 30,260,968 | 4.42 | 100 | 99.89 | 50 |
| FD4 | 43,745,290 | 40,402,008 | 5.93 | 100 | 99.89 | 50 |
| TD1 | 44,317,098 | 40,807,628 | 5.98 | 100 | 99.89 | 50 |
| TD2 | 43,798,146 | 40,681,472 | 5.97 | 100 | 99.89 | 50 |
| TD3 | 43,049,724 | 11,729,691 | 1.72 | 100 | 99.89 | 50 |
| TD4 | 43,897,326 | 40,794,108 | 5.98 | 100 | 99.89 | 50 |

**Table S2.** Unigenes of *Tuber indicum* annotated in different databases.

| **Database** | **Number of unigene** | **Percent (%)** |
| --- | --- | --- |
| NR | 93,555 | 55.05 |
| NT | 47,646 | 28.04 |
| KEGG | 25,358 | 14.92 |
| GO | 22,100 | 13.01 |
| SwissProt | 27,345 | 16.09 |
| KOG | 24,282 | 14.29 |
| Uniprot | 27,245 | 16.03 |
| Annotated in all databases | 11,425 | 6.72 |
| Annotated in at least one database | 98,839 | 58.16 |
| Total | 169,932 | 100 |

**Table S3**. GO annotation in three categories of GO terms, i.e., biological processes (BP), cellular component (CC), and molecular function (MF), respectively, based on unigenes identified in the transcriptome of *Tuber indicum* under 4 ºC.

| **GO term category** | **GO Term** | **Unigene count** |
| --- | --- | --- |
| BP | cellular process | 17,512 |
| BP | protein localization | 15,084 |
| BP | regulation of gene expression | 14,288 |
| BP | cellular protein metabolic process | 12,815 |
| BP | organelle organization | 12,570 |
| BP | nitrogen compound metabolic process | 12,314 |
| BP | RNA metabolic process | 11,415 |
| BP | gene expression | 11,308 |
| BP | nucleobase-containing compound metabolic process | 10,548 |
| BP | cellular protein modification process | 10,269 |
| BP | intracellular transport | 9,884 |
| BP | biological_process | 9,795 |
| BP | cellular protein localization | 9,720 |
| BP | regulation of transcription, DNA-templated | 9,696 |
| BP | RNA processing | 8,796 |
| BP | protein transport | 8,505 |
| BP | protein metabolic process | 8,385 |
| BP | translation | 8,180 |
| BP | negative regulation of gene expression | 8,080 |
| BP | regulation of transcription by RNA polymerase II | 8,080 |
| Total |  | 217,244 |
| CC | cytoplasm | 86,340 |
| CC | cell | 65,702 |
| CC | nucleus | 52,944 |
| CC | intracellular membrane-bounded organelle | 51,681 |
| CC | protein-containing complex | 39,231 |
| CC | intracellular | 27,555 |
| CC | membrane | 27,108 |
| CC | mitochondrion | 22,140 |
| CC | cytosol | 19,500 |
| CC | intracellular organelle | 15,906 |
| CC | integral component of membrane | 12,520 |
| CC | endoplasmic reticulum | 12,353 |
| CC | ribonucleoprotein complex | 12,024 |
| CC | plasma membrane | 9,900 |
| CC | intrinsic component of membrane | 9,624 |
| CC | cellular_component | 9,485 |
| CC | cell periphery | 7,908 |
| CC | endomembrane system | 7,616 |
| CC | membrane-bounded organelle | 7,468 |
| CC | chromosome | 7,110 |
| Total |  | 504,115 |
| MF | catalytic activity | 20,556 |
| MF | hydrolase activity | 14,721 |
| MF | protein binding | 13,741 |
| MF | nucleic acid binding | 11,202 |
| MF | transferase activity | 11,196 |
| MF | RNA binding | 11,052 |
| MF | molecular_function | 8,442 |
| MF | DNA binding | 7,540 |
| MF | drug binding | 5,691 |
| MF | organic cyclic compound binding | 5,574 |
| MF | ATPase activity | 5,480 |
| MF | ATP binding | 5,392 |
| MF | nucleotide binding | 5,195 |
| MF | enzyme binding | 5,040 |
| MF | oxidoreductase activity | 3,750 |
| MF | kinase activity | 3,736 |
| MF | structural molecule activity | 3,640 |
| MF | metal ion binding | 3,496 |
| MF | peptidase activity | 2,936 |
| MF | pyrophosphatase activity | 2,877 |
| Total |  | 151,257 |
